# Dataset on the comparison of synthesized and commercial zeolites for potential solar adsorption refrigerating system

**DOI:** 10.1016/j.dib.2018.07.040

**Published:** 2018-07-26

**Authors:** A.R. Sowunmi, C.O. Folayan, F.O. Anafi, O.A. Ajayi, N.O. Omisanya, D.O. Obada, D. Dodoo-Arhin

**Affiliations:** a Department of Mechanical Engineering, Ahmadu Bello University, Zaria, Nigeria; b Department of Chemical Engineering, Ahmadu Bello University, Zaria, Nigeria; c National Automotive Design and Development Council, Zaria, Nigeria; d National Universities Commission, 26, Aguiyi Ironsi Street, Maitama, Abuja, Nigeria; e Department of Material Science and Engineering, University of Ghana, Legon-Ghana; f Institute of Applied Science and Technology, University of Ghana, Legon-Ghana

## Abstract

The purpose of this dataset is to provide a comparison between synthesized and commercial 4A and 13X type zeolites. Metakaolin produced from the calcination of beneficiated kaolin at 750 °C for 4 h was dealuminated using sulphuric acid to get the required silica to alumina ratio for the zeolite synthesis. Zeolite 4A and 13X samples were characterized along-side with the commercial variants using X-ray fluorescence (XRF), X-ray diffraction (XRD), Brunauer, Emmett and Teller (BET) and scanning electron microscopy (SEM) techniques. These analyses revealed that, the zeolites synthesized are of comparatively acceptable quality. The pore size of 120.859 nm, pore volume of 0.0065 cm^3^/g and surface area of 22 m^2^/g were obtained from BET analyses for zeolite 4A synthesized from kaolin, while the commercial zeolite 4A used as control gave pore size of 58.143 nm, pore volume of 0.2462 cm^3^/g and surface area of 559.13 m^2^/g. In the same vein, the pore size of 10.5059 nm, pore volume of 0.135847 cm^3^/g and surface area of 324.584 m^2^/g were obtained from BET analyses for zeolite 13X synthesized from kaolin, while the commercial zeolite 13X gave pore size of 7.2752 nm, pore volume of 0.135951 cm^3^/g and surface area of 310.0906 m^2^/g.

**Specifications Table**

**Table:** 

Subject area	Engineering
More specific subject area	Adsorbents in a solar adsorption cooling system
Subject area	Tables and Figures
How data was acquired	XRF, XRD, SEM and BET techniques
Data format	raw values of XRF and BET, images and patterns of SEM and XRD
Experimental factors	1. Beneficiation to remove impurities from Kankara Kaolin. 2. Calcination of beneficiated kaolin at 750 °C for 4 h to produce metakaolin. 3. Dealumination of metakaolin 4. Gel formation and aging 5. Crystallization of aged product in an oven. 6. Analytical experimentation of synthesized and commercial 4A and 13X type zeolites
Experimental features	Metakaolin was produced from the calcination of beneficiated kaolin at 750 °C for 4 h and was dealuminated using sulphuric acid (96 wt/vol%) to get the required silica to alumina ratio for the zeolite synthesis.
Data source location	Department of Mechanical Engineering Ahmadu Bello University, Zaria, Nigeria.
Data accessibility	Data is available within this article

**Value of data**

● It is important to promote the local content, hence a need to compare zeolites synthesized from locally sourced clay and commercial zeolites. This will ascertain if local production of the zeolites is to be encouraged.
● The comparison of the zeolites (synthesized and commercial) in terms of the crystallinity and structure, elemental composition and morphology elucidated by XRD, XRF and SEM are of great importance.
● Pore sizes, volumes and specific surface areas further highlighted the potential application of the synthesized zeolites 4A and 13X as compared to the commercial zeolites 4A and 13X for continuous adsorption cooling systems.

## 1 Data

The dataset presented in this paper for XRD as shown in Fig. 2, Fig. 3 is the text file format plotted for the locally synthesized and commercial zeolites. The morphology of zeolites presented in Fig. 4, Fig. 5 is at a magnification of 10.0 kx for locally synthesized and commercial zeolites (4A and 13X). The XRF (Table 1, Table 2) and BET data (Table 3, Table 4) presented are elemental composition and surface area analysis results for locally synthesized and commercial zeolites respectively.

**Fig. 1:**
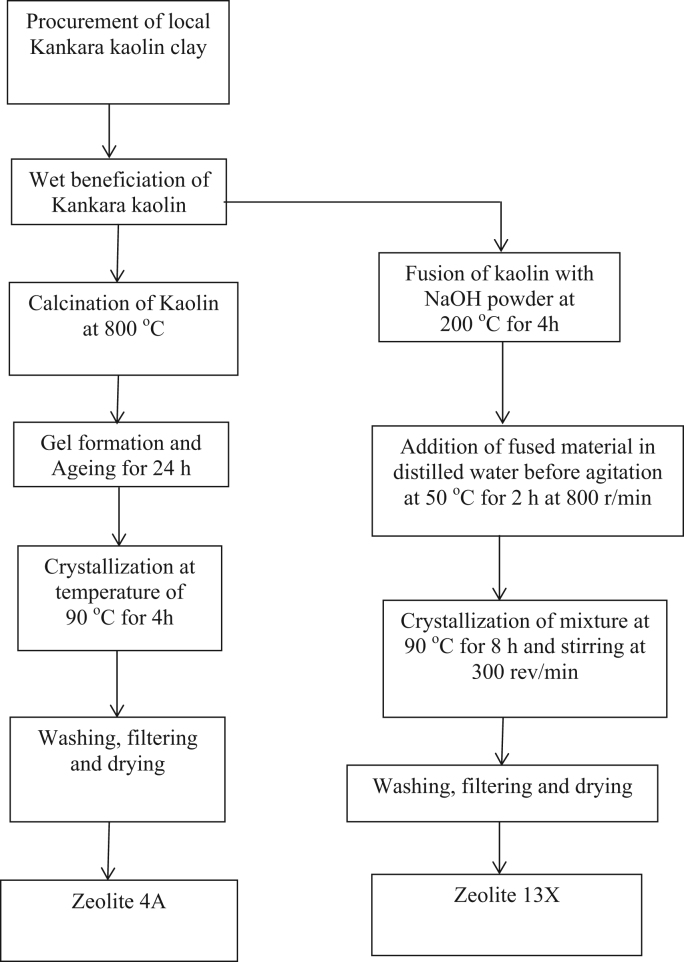
Flow diagram of the synthesis of Zeolites 4A and 13X from Kankara Kaolin.

**Fig. 2:**
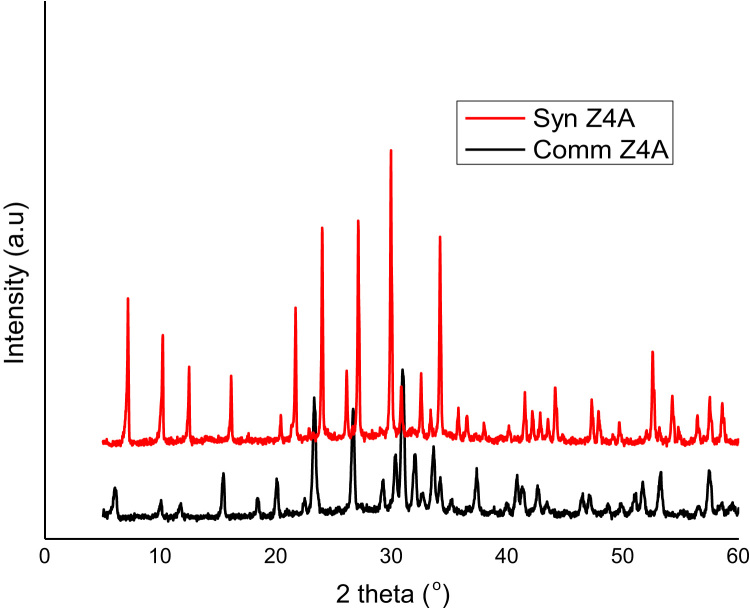
Comparative XRD spectra of zeolite 4A (synthesized and commercial).

**Fig. 3:**
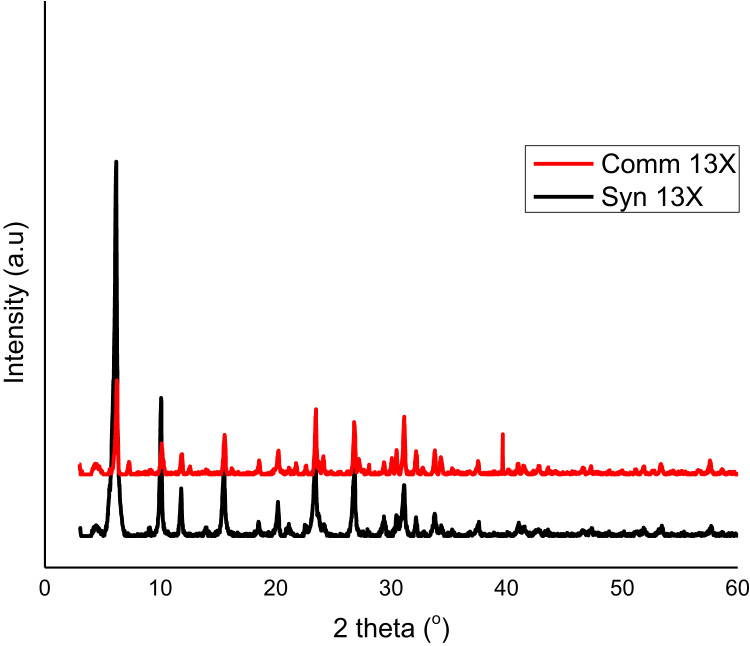
Comparative XRD spectra of zeolite 13X(synthesized and commercial).

**Fig. 4:**
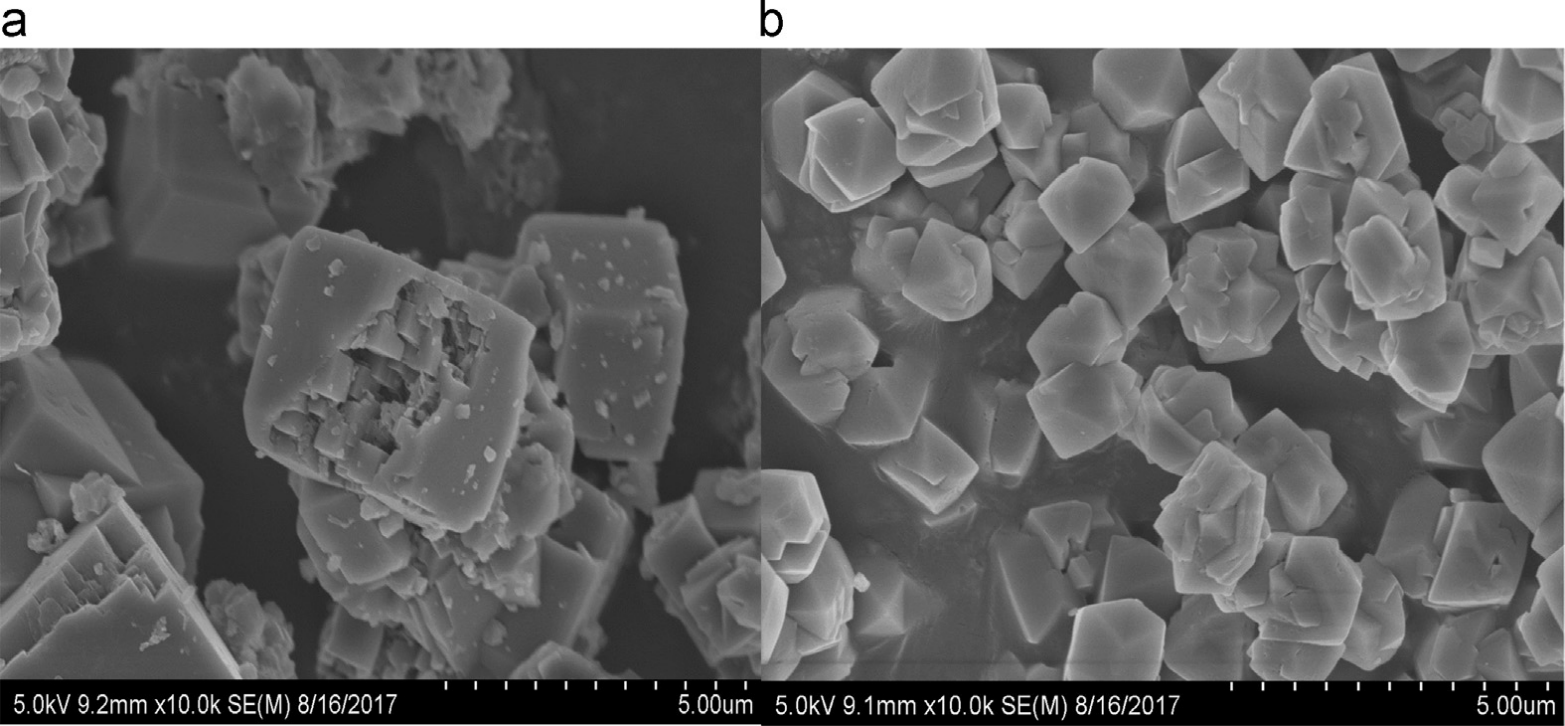
SEM micrograph of (a) zeolite 4A from Kankara kaolin (b) commercial zeolite 4A.

**Fig. 5:**
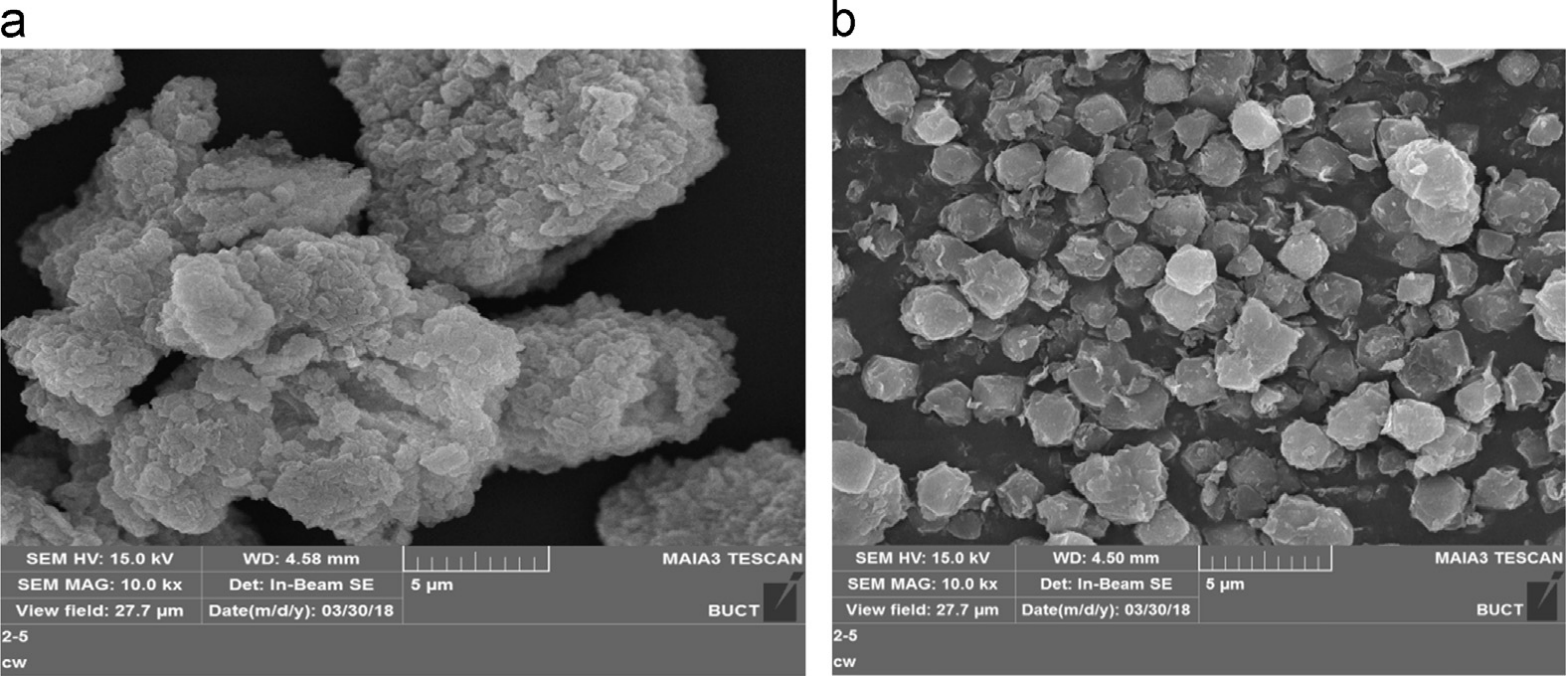
SEM micrograph of (a) zeolite 13X from Kankara kaolin (b) commercial zeolite 13X.

**Table 1:** Elemental composition of zeolite 4A from kaolin, commercial zeolite 4A and reference zeolite 4A.

Composition (wt%)	Syn 4A	Comm 4A	Reference 4A
Na_2_O	13.777	12.601	12.60
MgO	0.316	0.215	0.23
SiO_2_	49.719	55.695	35.90
Al_2_O_3_	34.673	30.645	31.19
K_2_O	0.187	0.239	0.400
TiO_2_	0.039	0.013	2.01
Fe_2_O_3_	0.664	0.059	1.13
**Si/Al**	2.440	3.090	2.000

Syn-synthesized; Comm-commercial; Reference zeolite 4A [3]; Si/Al – silica/alumina ratio.

**Table 2:** Elemental composition of zeolite 13X from kaolin, commercial zeolite 13X and reference zeolite 13X.

Composition (wt%)	Syn 13x	Comm 13X	Reference 13X
Na_2_O	9.757	15.930	12.49
MgO	0.502	0.8035	ND
SiO_2_	55.099	53.7975	49.28
Al_2_O_3_	30.180	28.580	30.17
K_2_O	0.400	0.0360	ND
TiO_2_	0.069	0.3660	ND
Fe_2_O_3_	0.836	2.080	ND
Si/Al	3.110	3.200	2.770

ND: Not detected; Syn-synthesized; Comm-commercial; Reference zeolite; Reference 13X – [4]. Si/Al – silica/alumina ratio.

**Table 3:** BET data of synthesized and commercial zeolite 4A.

BET analyses	Syn 4A	Comm 4A
Pore size (nm)	12.086	58.143
Specific surface area (m^2^/g)	22	559.13
Pore volume (cm^3^/g)	0.0065	0.2462

**Table 4:** BET data of synthesized and commercial zeolite 13X.

BET analyses	Syn 13X	Comm 13X
Pore size (nm)	10.5059	7.2752
Specific surface area (m^2^/g)	324.584	310.0906
Pore volume (cm^3^/g)	0.135847	0.135951

## 2 Experimental design, materials, and methods

A schematic of the experimental procedure of locally synthesized zeolite 4A and 13X is presented in Fig. 1 [1].

XRD patterns of the zeolites (synthesized and commercial) were collected on an Empyrean diffractometer (PANalytical BV, Netherlands) with theta/theta geometry, operating a Cu Kα radiation tube (*λ*=1.5418 Å) at 40 kV and 45 mA. The XRD patterns of all the randomly oriented powder specimens were recorded in the 5.0– 60° 2*θ* range with a step size of 0.017° and a counting time of 14 s per step. The surface morphology of the zeolites (synthesized and commercial; 4A and 13×) was carried out on an ultra-high vacuum and high resolution MAIA3 TESCAN scanning electron microscope operated at 15 kV. In addition, the zeolite samples were analyzed using a Micrometrics TriStar 3000 gas adsorption instrument which calculated the BET surface area. The surface area of the samples was measured by nitrogen adsorption at −196.15 °C. Prior to analyses, the samples were degassed in vacuum at 105 °C for 12 h. The specific surface area was calculated using the Brunauer-Emmett-Teller (BET) method [2].
