# Supplementary material for: Dataset on the comparison of synthesized and commercial zeolites for potential solar adsorption refrigerating system
Source: Data Brief. 2018 Jul 26;20:90–5. doi: 10.1016/j.dib.2018.07.040 (PMC6083001; doi:10.1016/j.dib.2018.07.040)
Supplement: Supplementary file 1 — Supplementary material [file mmc1.docx]

Mechanical Engineering Department,

Ahmadu Bello University,

Zaria, Nigeria.

11^th^ July, 2018

The Editor-in-Chief

DIB

Dear Sir,

**Submission of Revised Manuscript**

We, the authors of the manuscript titled “**Dataset on the comparison of synthesised and commercial zeolites for potential solar adsorption refrigerating system””** hereby wish to submit our revised manuscript for your kind attention.

We declare no conflict of interests.

Thank you and best regards.

Yours faithfully,


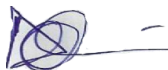


David O. Obada

For: Authors
